# The antidepressant impact of minocycline in rodents: A systematic review and meta-analysis

**DOI:** 10.1038/s41598-018-36507-9

**Published:** 2019-01-22

**Authors:** Daniel J. Reis, Emily J. Casteen, Stephen S. Ilardi

**Affiliations:** University of Kansas, Department of Psychology, Lawrence, KS 66045 USA

## Abstract

Evidence from recent animal studies suggest that minocycline, a broad-spectrum antibiotic capable of regulating immune processes, may possess antidepressant properties. These studies, however, have yet to be comprehensively reviewed. Accordingly, this systematic review and meta-analysis summarizes the extant literature examining the effect of minocycline on depressive-like behavior in rodent models. PubMed, PsycINFO, and Web of Science databases were systematically searched for articles that met prespecified inclusion and exclusion criteria, and standardized mean differences (SMDs) were calculated for each continuous measure of depressive-like behavior. The overall effect of minocycline on depressive-like behavior was estimated using robust variance estimation meta-analysis. Separate subgroup analyses were conducted on diseased vs healthy animal models, different rodent species, and immobility-based vs anhedonia-based measures of depressive-like behavior. A total of 22 preclinical studies (816 animals) were included. Overall, minocycline reduced depressive-like behavior in rodents (SMD = −1.07, 95% CI −1.41–−0.74, *p* < 0.001). Subgroup analyses revealed that minocycline reduced depressive-like behavior in diseased, but not healthy, animal models. Finally, minocycline was found to reduce both immobility-based and anhedonia-based outcomes. These findings suggest that minocycline may be an effective treatment of core depressive symptoms, and that further investigation of minocycline treatment for clinically relevant depression in humans is warranted.

## Introduction

Major depressive disorder is a debilitating form of mental illness that affects approximately 1 in 20 people worldwide^1,2^. The disorder is estimated to cost the United States over $200 billion per year in medical services and workplace-related losses^3^. Significantly, depression accounts for 7.5% of all years lived with disability worldwide—the greatest disability burden of any single disease^2^. It is also a significant source of mortality via depression-linked suicide, as well as through its association with ischemic heart disease^4^.

Antidepressant medications are often employed as the primary treatment for depressive illness. A recent comprehensive meta-analysis found 21 different antidepressants to be significantly more efficacious than placebo^5^, but the magnitude of this effect was rather modest for nearly all the drugs evaluated. Simply put, many depressed patients do not respond adequately to conventional antidepressant therapy^6–8^, and even those who do will typically face a high risk of relapse following acute treatment^6,9,10^. Such findings highlight the continued need for development of novel efficacious depression interventions.

Minocycline, a second-generation antibiotic with pronounced inhibitory effects on the brain’s microglia, may represent one such innovative approach. A growing body of evidence suggests that minocycline beneficially targets several pathways of relevance to the pathophysiology of depression. For instance, inflammation is strongly associated with depression^11,12^, and pro-inflammatory medications carry the potential to induce depressive symptoms in humans^13,14^. Preliminary evidence also suggests that some anti-inflammatory drugs may have antidepressant effects comparable to those of conventional pharmacological treatments^15^. Microglia are the primary immune cells within the central nervous system that mediate neuroinflammation^16^, and minocycline is capable of reducing inflammation both by directly suppressing microglia activation and by indirectly inhibiting microglia-induced inflammatory processes^17^. In doing so, minocycline also indirectly acts upon key monoaminergic systems implicated in depressive symptomatology, including the modulation of neurochemical cascades involving serotonin, dopamine, and norepinephrine^11^, as well as promotion of serotonin synthesis by reducing activation of the kynurenine pathway^17^. Finally, minocycline protects against oxidative stress and neuronal damage, two other harmful processes implicated in pathogenesis and maintenance of depression^17,18^.

Preliminary evidence also suggests that minocycline may have psychotropic effects in humans. The addition of minocycline to treatment with atypical antipsychotics has been shown to reduce negative symptoms, such blunted affect and anhedonia, in individuals with early-stage schizophrenia^19,20^. Additionally, an open-label study by Miyaoka *et al*.^21^ reported that minocycline, in combination with antidepressants, reduced depressive symptoms in individuals with psychotic depression. Only a few placebo-controlled clinical trials, however, have investigated minocycline as a treatment for depression in humans. Although a recent meta-analysis of three randomized controlled trials^22^ found support for an antidepressant effect of minocycline, it is premature to draw any substantive conclusions in light of the limited evidence available.

Despite the paucity of published clinical trials, there does exist a much larger preclinical (animal) literature regarding the antidepressant potential of minocycline. However, to the best of our knowledge, these preclinical studies have not yet been comprehensively reviewed. Accordingly, the purpose of this study is to summarize and quantify existing preclinical studies of the effect of minocycline on depressive behaviors by means of a systematic review and meta-analysis.

## Results

### Study selection and characteristics

**Figure 1:**
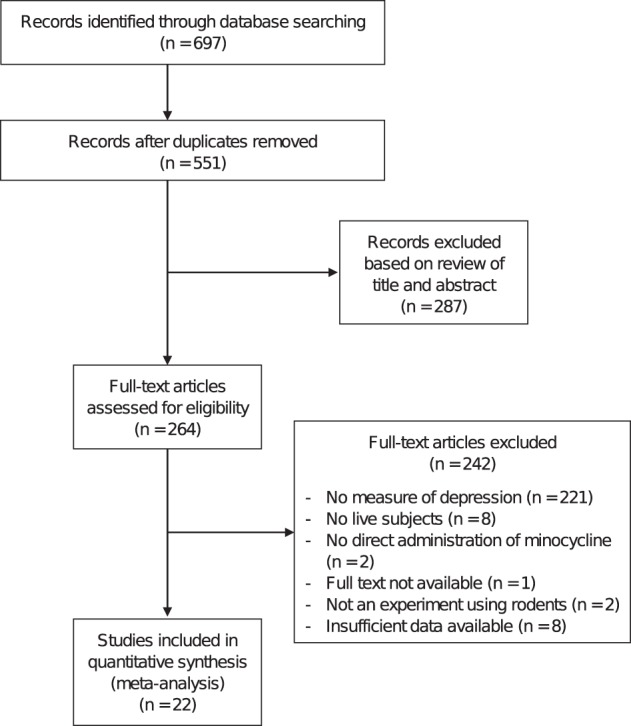
Flowchart of the selection of studies.

Twenty-two studies^23–44^ with 39 independent experimental groups and 816 rodent subjects were included in the meta-analysis (Fig. 1 and Table 1). Eleven studies used rats as experimental subjects and 11 others used mice. Twenty-five of the 39 experimental groups were identified as clinical models of disease, meaning that the animals were exposed to experimental manipulation in addition to their assigned treatment with either minocycline or placebo. Examples of experimental manipulation included chronic stress^25,30,37,39–41^, induced sickness^25,27–29,43^, olfactory bulbectomy-induced depression^24,34^, and induced type-1 diabetes^23^.

**Table 1:** Preclinical study characteristics.

Author, year	Subjects	No. of subjects	Days of intervention	Minocycline dosage	Depression Measure
Amorim, D. (2017)^23^	Male Wistar Han rats injected with streptozotocin	16	21	80 mg/kg	Forced swim test: ↔ latency to immobility; ↓ immobility
Burke, N. (2014) Group 1^24^	Male Sprague-Dawley rats subjected to olfactory bulbectomy	17	1	80 mg/kg	Open field test: ↔ locomotion
Burke, N. (2014) Group 2^24^	Male Sprague-Dawley rats subjected to olfactory bulbectomy	22	15	1 mg/ml of drinking water	Open field test: ↓ locomotion
Chijiwa, T. (2015) Group 1^25^	Male Wistar rats exposed to social defeat stress	11	5	50 mg/kg	Forced swim test: ↔ immobility
Chijiwa, T. (2015) Group 2^25^	Male Wistar rats exposed to social defeat stress and injected with polyl:C	12	5	50 mg/kg	Forced swim test: ↓ immobility
Deak, T. (2005)^26^	Male Sprague-Dawley rats	19	2	Low dose - 20 mg/kg; High dose - 40 mg/kg	Forced swim test: ↔ immobility
Henry, C. (2008) Group 1^27^	Male BALB/c mice	30	3	50 mg/kg	Sucrose preference test: ↔ % preference
Henry, C. (2008) Group 2^27^	Male BALB/c mice injected with *Escherichia coli* (LPS)	30	3	50 mg/kg	Sucrose preference test: ↑ % preference
Mahmoud, M. (2017) Group 1^28^	Female BALB/c mice	12	4	10 mg/kg	Forced swim test: ↔ immobility; Sucrose preference test: ↔ % preference
Mahmoud, M. (2017) Group 2^28^	Female BALB/c mice infected with *Toxoplasma gondii*	11	4	10 mg/kg	Forced swim test: ↓ immobility; Sucrose preference test: ↔ % preference
Majidi, J. (2016) Group 1^29^	Male NMRI neonatal mice	18	35	30 mg/kg	Forced swim test: ↔ immobility; Sucrose preference test: ↔ % preference; Tail suspension test: ↔ immobility
Majidi, J. (2016) Group 2^29^	Male NMRI neonatal mice injected with *Escherichia coli* (LPS)	18	35	30 mg/kg	Forced swim test: ↓ immobility; Sucrose preference test: ↑ % preference; Tail suspension test: ↓ immobility
McKim, D. (2016)^30^	Male C57BL/6 mice subjected to repeated social defeat	21	6	90 mg/kg	Repeated social defeat model: ↔ time in interaction zone
Molina-Hernandez, M. (2008)^31^	Male Wistar rat	28	1 (3 injections)	Low dose - 50 mg/kg; medium dose - 60 mg/kg; high dose - 80 mg/kg	Forced swim test: ↓ immobility
Molina-Hernandez, M. (2008)^32^	Male Wistar rat	21	1 (3 injections)	Low dose - 50 mg/kg; high dose - 60 mg/kg	Forced swim test: ↓ immobility
Nagpal, K. (2013)^33^	Male Albino Mice	18	1	100 mg/kg	Forced swim test: ↓ immobility; Tail suspension test: ↓ immobility
Rinwa, P. (2013) Group 1^34^	Male Wistar rat subjected to olfactory bulbectomy	36	14	Low dose - 25 mg/kg; High dose -50 mg/kg	Forced swim test: ↓ immobility; Open field test: ↓ locomotion (# of sections crossed)
Rinwa, P. (2013) Group 2^34^	Male Wistar rat subjected to olfactory bulbectomy and treated with quercetin (low dose)	24	14	25 mg/kg	Forced swim test: ↓ immobility; Open field test: ↓ locomotion (# of sections crossed)
Rinwa, P. (2013) Group 3^34^	Male Wistar rat subjected to olfactory bulbectomy and treated with quercetin (medium dose)	24	14	25 mg/kg	Forced swim test: ↓ immobility; Open field test: ↓ locomotion (# of sections crossed)
Saravi, S. (2016)^35^	Male Wistar rat subjected to testicular torsion	24	1	Low dose - 40 mg/kg; Medium dose - 80 mg/kg; High dose - 160 mg/kg	Forced swim test: ↓ immobility
Saravi, S. (2016)^36^	Male NMRI mice given the pesticide malathion	24	1	Low dose - 40 mg/kg; Medium dose - 80 mg/kg; High dose - 160 mg/kg	Forced swim test: ↓ immobility; Tail suspension test: ↓ immobility
Singh, B. (2017)^37^	Male albino LACA mice subjected to chronic restraint stress	12	30	100 mg/kg	Forced swim test: ↓ immobility
Singh, T. (2016) Group 1^38^	Male swiss albino mice subjected to kindling-induced epilepsy	12	15	40 mg/kg	Forced swim test: ↓ immobility; Tail suspension test: ↓ immobility
Singh, T. (2016) Group 2^38^	Male swiss albino mice subjected to kindling-induced epilepsy and treated with valproate	24	15	Low dose - 10 mg/kg; Medium dose - 20 mg/kg; High dose - 40 mg/kg	Forced swim test: ↔ immobility; Tail suspension test: ↔ immobility
Tong, L. (2017) Group 1^39^	Male ICR Mice	24	42	40 mg/kg	Forced swim test: ↔ immobility; Sucrose preference test: ↔ % preference; Tail suspension test: ↔ immobility
Tong, L. (2017) Group 2^39^	Male ICR Mice subjected to chronic unpredictable stress	24	42	40 mg/kg	Forced swim test: ↓ immobility; Sucrose preference test: ↑ % preference; Tail suspension test: ↓ immobility
Tong, L. (2017) Group 3^39^	Male ICR Mice	24	28	40 mg/kg	Forced swim test: ↔ immobility; Sucrose preference test: ↔ % preference; Tail suspension test: ↔ immobility
Tong, L. (2017) Group 4^39^	Male ICR Mice subjected to chronic restraint stress	24	28	40 mg/kg	Forced swim test: ↓ immobility; Sucrose preference test: ↑ % preference; Tail suspension test: ↓ immobility
Tong, L. (2017) Group 5^39^	Male ICR Mice	24	20	40 mg/kg	Forced swim test: ↔ immobility; Sucrose preference test: % ↔ preference; Tail suspension test: ↔ immobility
Tong, L. (2017) Group 6^39^	Male ICR Mice subjected to chronic social defeat stress	24	20	40 mg/kg	Forced swim test: ↓ immobility; Sucrose preference test: ↑ % preference; Tail suspension test: ↓ immobility
Wang, H.-T. (2017) Group 1^40^	Male Sprague-Dawley rats	16	7	50 mg/kg	Forced swim test: ↔ immobility; Sucrose preference: ↔ % preference
Wang, H.-T. (2017) Group 2^40^	Male Sprague-Dawley rats subjected to early-life social isolation	18	7	50 mg/kg	Forced swim test: ↓ immobility; Sucrose preference test: ↑ % preference
Wong, M.-L. (2016) Group 1^41^	Male C57BL mice	24	21	5 mg/kg	Forced swim test: ↔ immobility
Wong, M.-L. (2016) Group 2^41^	Male C57BL mice subjected to chronic restraint stress	30	21	5 mg/kg	Forced swim test: ↔ immobility
Xu, N. (2017) Group 1^42^	Male Sprague-Dawley rats	24	15	40 mg/kg	Forced swim test: ↔ immobility; Sucrose preference test: ↔ % preference
Xu, N. (2017) Group 2^42^	Male Sprague-Dawley rats subjected to spared nerve injury	24	15	40 mg/kg	Forced swim test: ↓ immobility; Sucrose preference test: ↑ % preference
Zheng, L.-S. (2015) Group 1^43^	Male C57BL/6 J mice	20	37	50 mg/kg	Forced swim test: ↔ immobility; Tail suspension test: ↔ immobility
Zheng, L.-S. (2015) Group 2^43^	Male C57BL/6 J mice injected with interferon-alpha	20	37	50 mg/kg	Forced swim test: ↓ immobility; Tail suspension test: ↓ immobility
Zheng, X. (2014)^44^	Male Wistar rats injected with *Escherichia coli* (LPS)	12	3	30 mg/kg	Sucrose preference test: ↑ % preference

↓ and ↑ represent a statistically significant decrease or increase (respectively) in measured behavior in at least one treatment group, while ↔ represents a nonsignificant or unclear change.

In total, 17 studies assessed immobility-based depressive-like behavior with either the forced swim test or the tail-suspension test, while seven studies assessed anhedonia-based depressive-like behavior with the sucrose preference test (and five studies used both immobility-based and anhedonia-based measures). Two studies assessed olfactory bulbectomy-induced hyperactivity with the open field test, proposed to be a measure of depressive-like behavior in this specific rodent model^24,34^. Finally, one study assessed social avoidance.

Doses of minocycline ranged from 10–160 mg/kg and the duration of treatment ranged from a single administration to 42 consecutive days. Minocycline was most commonly administered by either intraperitoneal injection or through drinking water.

### Bias assessment

Risk of bias assessment for the included studies can be viewed in Table 2. Almost no study provided sufficient detail regarding selection, performance, or detection bias. Poor reporting of experimental and statistical methods is a recognized problem in animal research that negatively affects the validity and reproducibility of published findings^45^. More detail was generally provided regarding attrition, reporting, and other forms of potential bias. Over half of the included studies had a low risk of attrition and other bias, while all included studies had a low risk of reporting bias. One study had a high risk of detection bias^41^, and five studies had a high risk of other bias^29,30,34,38,42^. A separate sensitivity analysis revealed that removal of these studies did not substantively impact our final results. Overall, however, the risk of bias for each individual study is unclear, which suggests that the observed treatment effects may be overestimated^46,47^.

**Table 2:** Risk of bias assessment.

Study	Sequence generation	Baseline characteristics	Blinding (intervention)	Incomplete outcome data	Selective reporting	Other bias
Amorim *et al*.^23^	Unclear	Low	Unclear	Unclear	Low	Low
Burke *et al*.^24^	Unclear	Unclear	Unclear	Unclear	Low	Low
Chijiwa *et al*.^25^	Unclear	Unclear	Unclear	Low	Low	Low
Deak *et al*.^26^	Unclear	Unclear	Unclear	Low	Low	Low
Henry *et al*.^27^	Unclear	Unclear	Unclear	Low	Low	Low
Mahmoud *et al*.^28^	Unclear	Unclear	Unclear	Unclear	Low	Low
Majidi *et al*.^29^	Unclear	Unclear	Unclear	Unclear	Low	High
McKim *et al*.^30^	Unclear	Unclear	Unclear	Low	Low	High
Molina-Hernandez *et al*.^31^	Unclear	Unclear	Unclear	Low	Low	Low
Molina-Hernandez *et al*.^32^	Unclear	Unclear	Unclear	Low	Low	Low
Nagpal *et al*.^33^	Unclear	Unclear	Unclear	Low	Low	Low
Rinwa & Kumar^34^	Unclear	Unclear	Unclear	Low	Low	High*
Saravi *et al*.^35^	Unclear	Unclear	Unclear	Low	Low	Low
Saravi *et al*.^36^	Unclear	Unclear	Unclear	Unclear	Low	Low
Singh *et al*.^37^	Unclear	Unclear	Unclear	Unclear	Low	Low
Singh & Goel^38^	Unclear	Unclear	Unclear	Low	Low	High*
Tong *et al*.^39^	Unclear	Unclear	Unclear	Low	Low	Low
Wang *et al*.^40^	Unclear	Unclear	Unclear	Unclear	Low	Low
Wong *et al*.^41^	Low	Unclear	High	Low	Low	Low
Xu *et al*.^42^	Unclear	Unclear	Unclear	Low	Low	High
Zheng *et al*.^43^	Unclear	Unclear	Unclear	Unclear	Low	Low
Zheng *et al*.^44^	Unclear	Unclear	Unclear	Low	Low	Low

Risk of bias relating to Allocation concealment, Random housing, Blinding (assessment), and Random outcome assessment was unclear for all included studies, and as such these domains have been omitted from the table. *High risk of bias only for the treatment groups receiving an intervention in addition to minocycline. For the treatment groups receiving only minocycline, risk of bias was low.

### Minocycline efficacy

**Figure 2:**
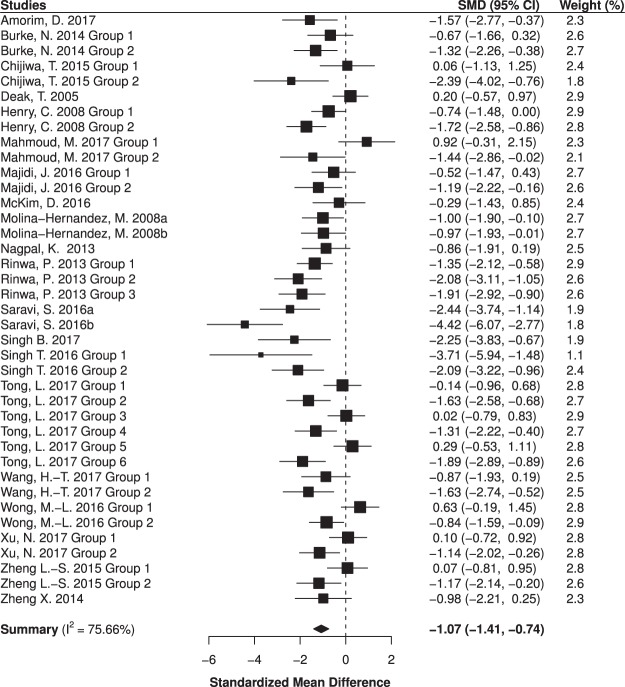
Meta-analysis of studies investigating the antidepressant effect of minocycline in rodents. SMD = Standardized mean difference; CI = Confidence interval. An aggregate SMD is displayed for each experimental group. A measure-specific forest plot can be viewed in Supplementary Fig. S1.

Combining standardized mean differences (SMDs) for the 39 included experimental groups revealed a pooled SMD of −1.07 (95% CI −1.41–−0.74, *p* < 0.001; Fig. 2). Minocycline administration, compared to placebo, significantly reduced depressive-like behavior in rodents. This effect was not significantly moderated in meta-regressions by either sample size (β = −0.001, 95% CI: −0.06–0.05, *p* = 0.961), treatment duration (β = 0.01, 95% CI: −0.01–0.04, *p* = 0.375), or treatment dose (β = −0.02, 95% CI: −0.03–0.003, *p* = 0.091).

Subgroup analyses revealed that minocycline administration significantly reduced depressive-like behavior only among diseased animals (SMD = −1.6, 95% CI: −1.93 –−1.26, *p* < 0.001); it had no significant impact among healthy animals, i.e., those naïve to experimental manipulation (SMD = −0.21, 95% CI: −0.55–0.14, *p* = 0.221). Minocycline also significantly reduced depressive-like behavior in both rats (SMD = −1.22, 95% CI: −1.71 –−0.72, *p* < 0.001) and mice (SMD = −0.97, 95% CI: −1.44 –−0.49, *p* < 0.001). Separate analyses revealed that minocycline significantly reduced both immobility-based (SMD = −1.17, 95% CI: −1.56 –−0.77, *p* < 0.001) and anhedonia-based (SMD = −0.78, 95% CI: −1.28 –−0.27, *p* = 0.005) outcomes. An exploratory meta-regression indicated that both minocycline pretreatment and acute treatment in diseased animals resulted in a significant reduction in depressive-like behavior compared to healthy animals (β = −0.91, 95% CI: −1.51 –−0.32, *p* = 0.005 and β = −1.64, 95% CI: −2.15 – −1.12, *p* < 0.001, respectively). Additionally, treatment following disease induction was associated with a significantly greater antidepressant effect than that of pretreatment (β = −0.72, 95% CI: −1.35 –−0.09, *p* = 0.027).

### Publication bias and heterogeneity

**Figure 3:**
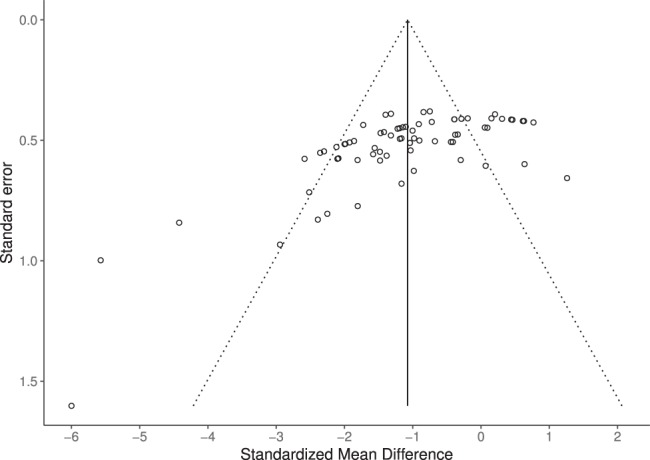
Funnel plot of standardized mean differences.

Visual inspection of a funnel plot (Fig. 3) revealed the presence of potential asymmetry, which was further supported by the use of an Egger test (*t* = −5.92, *df* = 69, *p* < 0.001). Together, these findings suggest that publication bias may be present in the included data, resulting in the omission of non-significant or opposing findings in the final analyses. There was also high heterogeneity across the 39 experimental groups (I^2^ = 75.7%), indicating a great deal of inconsistency across studies. High heterogeneity and funnel plot asymmetry, which have been previously identified as a significant problem among meta-analyses of animal research, weaken the generalizability of overall findings^48^. Inclusion of study characteristics, such as sample size, treatment differences, and subject species/strain as moderating variables failed to reduce heterogeneity. However, separate analysis of diseased and healthy animals did reduce heterogeneity to moderate levels (I^2^ = 61.6% and I^2^ = 55.7%, respectively), supporting recent proposals that the use of consistent and well-validated experimental models may aid in the translation of preclinical research findings^49^.

## Discussion

This systematic review and meta-analysis included 22 preclinical studies, which encompassed a total of 39 independent experimental groups and 816 rodents. Minocycline administration significantly decreased depressive-like behavior among study animals in our primary analysis, with a robust pooled standardized mean difference (SMD) of −1.07 in comparison with placebo. Moreover, 19 of the 22 included studies reported a statistically significant minocycline effect on at least one salient outcome measure.

Notably, however, minocycline only evidenced significant antidepressant effects among animals that were already experimentally stressed or diseased before they faced the depression assessment protocol—typically another acutely stressful procedure such as forced swimming or prolonged tail suspension. The drug had no significant impact upon animals that entered the challenging depression paradigm in an otherwise healthy state. This striking between-group contrast may perhaps best be explained by critical differences in depression symptom severity among the healthy and diseased rodents, as the induced disease states among study animals—chronic stress^30,37,39^, provoked inflammation or sickness^25,27,29^, and olfactory bulbectomy^24,34^—are all associated with an increase in depressive-like behavior^23–25,27,37,50,51^. In other words, it is possible that minocycline only exerts significant antidepressant effects in the presence of elevated levels of depressive symptomatology. This conjecture is congruent with the finding that conventional antidepressants outperform placebo most consistently in more severe cases of depression^52,53^. Accordingly, future clinical trials might be advised to attend carefully to baseline depression severity among study patients, and perhaps to focus the investigation of minocycline effects upon the *severe* and *very severe* depression subtypes.

In our secondary analyses, minocycline reduced depressive-like behavior in diseased animals regardless of whether the drug was administered as a pretreatment—prior to depressogenic challenge—or as an acute treatment of existing symptomatology. Such a finding has clear parallels in the clinical literature. Although antidepressants are most commonly prescribed to treat active depressive episodes^54^, they also seem to protect against the onset of iatrogenic (treatment-induced) depression in humans. For example, prophylactic antidepressant use reduces the risk of depression among patients receiving depressogenic interferon-α treatment^55^. Additionally, patients administered an antidepressant prior to surgery or radiation therapy for cancer may become less likely to develop depression during treatment^56^. Because minocycline pretreatment likewise protects against disease-induced depressive-like behavior in rodents, it will be valuable to see future investigation of its prophylactic value in humans, especially for medically-induced depression in clinical settings. On the other hand, we did find that the therapeutic effect of minocycline was largest when administered *following* disease induction, which raises the possibility that the drug might be most useful as a treatment for *existing* depressive symptoms.

It is also worth noting that minocycline improved performance on both immobility- and anhedonia-based measures of depressive-like behavior in study analyses. Immobility measures, such as the forced swim test and the tail suspension test, are widely used to screen potential antidepressants, and they are considered to represent negative affectivity^50,51,57^, although there is still some debate over the extent to which immobility challenge behaviors reflect a true depressive phenotype^58,59^. The sucrose preference test, on the other hand, is used to assess anhedonic behaviors^59^. Negative mood and anhedonia are fundamental features of depression; in fact, the current classification of major depressive disorder effectively requires at least one of these two symptoms to be present for diagnosis^60^. Anhedonia and dysphoria also both contribute substantially to psychosocial impairment in depression^61^, rendering them particularly important targets for antidepressant therapy. Inasmuch as minocycline may address both symptom domains, it may have potentially far-reaching clinical utility.

The observed antidepressant-like effects of minocycline may derive in part from the drug’s established anti-inflammatory properties, including its ability to inhibit microglia-induced inflammatory processes in the brain. In our analysis, five of six studies found that minocycline had a meaningful effect on depressive-like behavior after the experimental induction of an inflammatory state^25,27–29,43,44^. Minocycline was also observed either to alter microglia function, including glial gene expression, or to reduce biomarkers of inflammation in 10 studies^24,27–29,34,37,39,40,42,43^. Finally, one reviewed study found that minocycline decreased depressive-like behavior while simultaneously altering the composition of the gut microbiota^41^—symbiotic microorganisms that play a key role in regulating immune processes^62^—thereby highlighting another possible route by which minocycline could target inflammation.

One important interpretive caveat for the present work is the fact that the risk of bias for each reviewed study is unclear—a pervasive, well-documented limitation of much preclinical research^63^. Insufficient detail was provided for seven of the 10 domains enumerated in the influential SYRCLE risk-of-bias tool^64^, thereby raising concerns that observed results may be affected by biases related specifically to inadequate randomization, allocation concealment, and blinding procedures. Such biases can lead to an overestimation of treatment effects^46,47^, which can in turn hamper translational research efforts. In fact, only an estimated 10% of investigational drugs that begin clinical testing obtain final approval, and those that reach later phases of clinical trials frequently fail due to lack of efficacy^65^. Reducing bias in preclinical trials can help to decrease such attrition by facilitating more accurate predictions of clinical efficacy^49^, which may in turn decrease the financial and human burden associated with failed clinical trials. Future investigations of minocycline, accordingly, should take care to properly implement and report procedures to reduce selection, performance, and detection bias.

A major barrier facing the use of minocycline in clinical settings is acquired resistance. Similar to other forms of antibiotic, minocycline usage is linked to the development of resistant bacterial strains^66^. It is possible that such resistance might diminish the antidepressive efficacy of minocycline, even if not used in an antibacterial capacity. Likewise, it may be useful to investigate the degree to which adjuvant medications designed to combat acquired resistance, such as loperamide^67^, potentiate the effects of minocycline in depressed patients.

One important limitation of the present analysis concerns the high level of methodological heterogeneity present in the reviewed studies. Subject characteristics, animal models, treatment dose, and administration period differed significantly across studies. Because high heterogeneity reduces the predictive validity of meta-analyses^48,68^, the present results should be interpreted with appropriate caution. A major source of this heterogeneity was simply the methodological variability across studies, as the mere separation of healthy and diseased animals for analytic purposes led to a substantial reduction in statistical heterogeneity.

Another limitation of the present work is the fact that sample sizes of included studies typically ranged between 10 to 30 animals. Such small sample sizes, common in preclinical studies, can negatively affect both the reliability and validity of study outcomes^69^. They can also lead to publication bias, which our analyses suggest may be present in the minocycline preclinical literature; if so, it could further weaken the generalizability of study results^69^. Also, although 39 experimental groups—a reasonably large number for meta-analytic purposes—were included in the overall analysis, the number was roughly halved for our two key subgroup analyses (diseased vs. healthy animals and immobility vs. anhedonia measures). Because subgroup analysis can result in substantially reduced statistical power^70^, this study may not have been adequately powered to detect all statistically significant subgroup effects. Finally, our search strategy, limited to the inclusion of only mice and rat subjects and English-language articles, may have introduced bias^71^.

In conclusion, minocycline was observed to significantly reduce depressive-like behavior on both immobility- and anhedonia-based measures. These findings support the desirability of additional clinical trials to investigate the antidepressant potential of this antibiotic among humans. Because minocycline had no effect on depressive-like behavior in healthy animals, it may prove optimal for future research to focus principally upon the treatment of clinically significant depression, as opposed to prophylaxis among healthy individuals. Finally, the anti-inflammatory effects of minocycline should be more extensively investigated as a promising candidate mechanism of antidepressant action.

## Methods

We adhered to CAMARADES guidelines for conducting systematic reviews and meta-analyses of animal studies^72^. The study protocol was not preregistered and can be viewed at 10.17504/protocols.io.vege3bw.

### Criteria

Studies were deemed eligible if they met the following inclusion criteria: 1) study subjects were either rats or mice; 2) minocycline was experimentally administered; 3) depressive-like behavior was a primary or secondary study outcome.

Studies were omitted from review if they met one or more of the following exclusion criteria: 1) there was no matched control group; 2) the experimental subjects did not directly receive the administration of minocycline (e.g., the treatment was administered to the subjects’ mothers); 3) descriptive outcome data (means, standard deviations, and sample sizes) were not available for the measured depressive-like behavior; 4) the full text of the article was not available in English. Conference abstracts were omitted due to lack of necessary information.

### Search strategy

Potential studies were identified by searching PubMed, PsycINFO, and Web of Science databases, from the earliest record of the databases to September 2017. Search terms included minocycline OR probiotic AND depressive-like (the exemplar PubMed search strategy can be found in the Supplementary Information). All studies were screened and evaluated in a standardized manner by two independent reviewers (DR and EC). First, the title and abstract for each search result were reviewed to identify potential studies, after which full-texts were evaluated to determine study inclusion. Disagreements between reviewers were resolved by discussion, with final decisions made by consensus. The flow chart of study selection can be viewed in Fig. 1.

### Data collection

Data collection was conducted using a custom form based on CAMARADES guidelines^72^. Extracted data included subject information (e.g., rodent species and strain, age, experimental status), intervention type (e.g., minocycline dose and duration), and outcome data (e.g., outcome measure, sample sizes, means and variances). Different measures of depressive-like behavior were assumed to be equivalent. If depressive-like behavior was measured at multiple time points throughout the study, the final measurement of the interventional period was selected as the included data. Data presented only in graphical format were extracted using WebPlotDigitizer graph digitization software^73^, a program recommended for use in systematic reviews^74^. Potential study bias was evaluated using SYRCLE’s risk-of-bias tool^64^. All data and risk-of-bias evaluations were extracted by two independent reviewers (DR and EC), and disagreements during this process were resolved by discussion. Finally, 11 authors were contacted and asked to provide additional study details and data. Two authors responded and these studies were included in the final study selection. Extracted study characteristics can be viewed in Table 1.

### Statistical analyses

The meta-analyses were performed with R 3.4.4 software^75^. All analyses were pre-specified unless otherwise stated. A standardized mean difference (SMD; also known as Hedges’ g) between minocycline-treated and matched control groups was calculated for all continuous measures of depressive-like behavior. A standard normal distribution was used to calculate confidence intervals for each SMD. Sample size, treatment duration, and treatment dose were assed as moderating variables in individual meta-regressions—study-level variables, rather than aggregated patient-level data, were chosen as moderators to avoid potential ecological bias^76^. Separate subgroup analyses were conducted on diseased (those receiving additional experimental manipulations prior to outcome measurement, such as induced sickness, chronic stress, etc.) and healthy animals (those receiving only minocycline or placebo intervention prior to outcome measurement), as well as mouse and rat samples. An exploratory meta-regression was conducted to compare minocycline effects, respectively, among healthy animals, diseased animals given the drug prior to disease induction (pretreatment), and diseased animals given minocycline after disease induction (treatment). Results from immobility-based (i.e. forced swim test and tail suspension test) and anhedonia-based (i.e. sucrose preference test) measures of depressive-like behavior were also analyzed in separate analyses. Multiple rodent subgroups within a single study (e.g., different rodent strains or experimental conditions) were included as independent SMDs, provided that each treatment group had a separate, matched control group. Whenever multiple treatment groups were compared against the same control group, an SMD was calculated from the combined results of the different treatment groups^77^.

If multiple measures of depressive-like behavior were available for a single treatment group, then a separate SMD was calculated for each outcome. Summary SMDs were calculated using robust variance estimation (RVE) meta-analyses, a form of random-effects meta-analysis shown to address dependency between SMDs measured in the same sample^78^. In RVE meta-analysis, multiple outcomes from a single study are included as separate SMDs and share a single study weight. SMDs were weighted by their precision (i.e., inverse variance). Between-study heterogeneity was evaluated using the I^2^ statistic, with values of I^2^ more than 25%, 50%, and 75% selected to reflect low, moderate, and high heterogeneity, respectively^79^. Potential publication bias was assessed for using funnel plot asymmetry and an Egger test^80^.

## Electronic supplementary material

Supplementary Information

Dataset 1

## Data Availability

All data analyzed during this study are included in this article and its Supplementary Information files.
