# Supplementary material for: The antidepressant impact of minocycline in rodents: A systematic review and meta-analysis
Source: Sci Rep. 2019 Jan 22;9:261. doi: 10.1038/s41598-018-36507-9 (PMC6342970; doi:10.1038/s41598-018-36507-9)

The antidepressant impact of minocycline in rodents: A systematic review and meta-analysis

Daniel J. Reis[1,*,+], Emily J. Casteen[1,+], and Stephen S. Ilardi[1]

[1]University of Kansas, Department of Psychology, Lawrence, KS, 66045, USA

* daniel.reis@ku.edu

[+]These authors contributed equally to this work

PubMed Search Algorithm

Step	Search Term
1	Rat* or mouse* or mice* or murine or rodent
2	Minocycline* or Minocin*
3	1 and 2
4	Depression or Depressed or dep* or depressive-like or depression-like
5	Antidepressant or Antidepressant-like
6	Antibiotic
7	mental health*
8	Psycholo* and stress
9	4 or 5 or 6 or 7 or 8
10	3 and 9

Variable Dictionary for Supplementary Data

Variable	Description
study	Study number
author	Author name
year	Year of publication
tx_dur	Duration of minocycline treatment in days
tx_dose	Minocycline treatment dose in billions of milligrams per kilogram
tx_n	Sample size of treatment group
c_n	Sample size of control group
hedges	Standardized mean difference between treatment and control group (Hedge's g) – Negative value = greater depression reduction in treatment group
se	Standard error of standardized mean difference
paradigm	Behavioral paradigm
measure	Specific paradigm measure to assess for depressive-like behavior
rodent	Rodent species employed – mouse or rat
disease	Animal model of disease (0 = no, 1 = yes)
dis_pre	Minocycline treatment prior to disease induction (0 = no, 1 = yes; note – 0 for both dis_pre and dis_tre = healthy animal)
dis_tre	Minocycline treatment following disease induction (0 = no, 1 = yes; note – 0 for both dis_pre and dis_tre = healthy animal)

Supplementary Figure S1

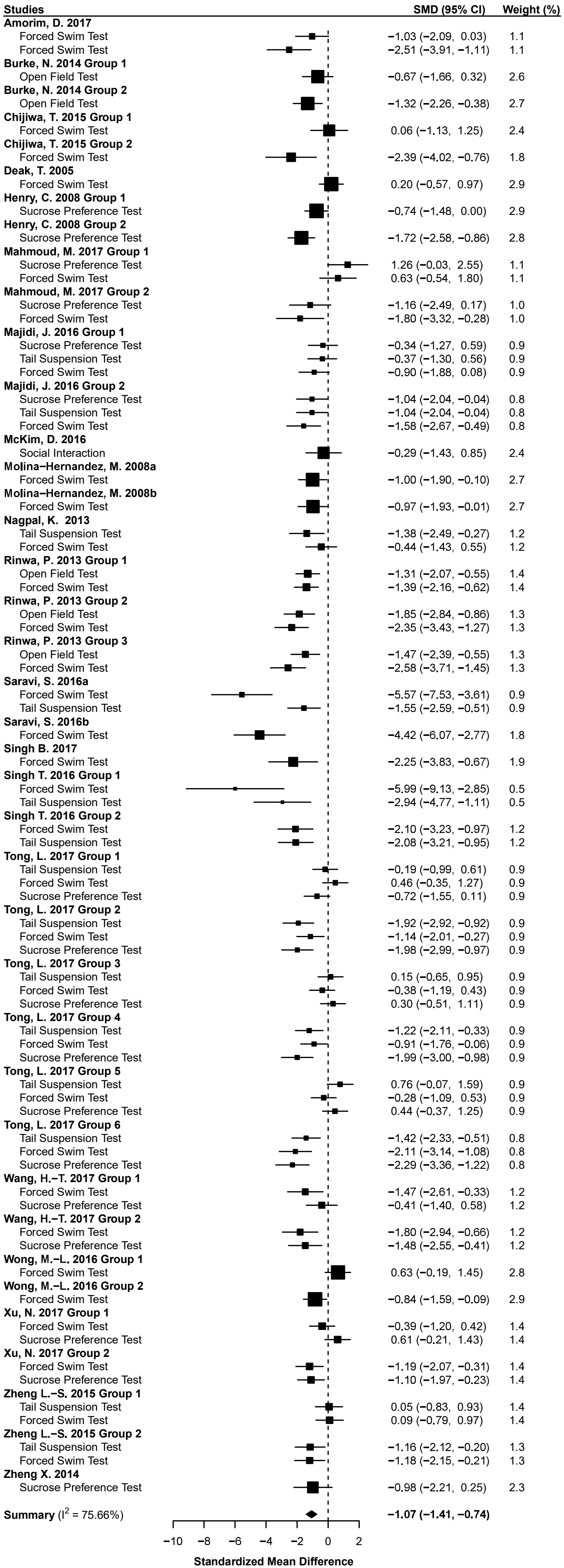

Supplement: Supplementary file 1 — Supplementary Information [file 41598_2018_36507_MOESM1_ESM.pdf]
